# An Unusual Neck Mass: A Case of a Parathyroid Cyst and Review of the Literature

**DOI:** 10.1155/2015/243527

**Published:** 2015-05-07

**Authors:** Anand Goomany, Amy Rafferty, Ian Smith

**Affiliations:** Bradford Royal Infirmary, Duckworth Lane, Bradford BD9 6RJ, UK

## Abstract

Parathyroid cysts (PC) are an unusual cause of neck swellings. The majority are nonfunctioning and prove to be a diagnostic challenge given their nonspecific physical and radiological characteristics. This is compounded by their rare occurrence, leading them to be overlooked in the differential diagnosis of neck lumps. Imaging techniques fail to determine the origin of these lesions, but a preoperative diagnosis can be achieved by fine-needle aspiration and measurement of cystic fluid C-terminal parathyroid hormone levels. Treatment of nonfunctioning cysts remains controversial and includes needle aspiration, injection of sclerosant, or surgical excision. We present a case of a 44-year-old female presenting with an asymptomatic anterior neck swelling, diagnosed postoperatively as a parathyroid cyst.

## 1. Introduction

Neck lumps are a common presenting complaint to the otolaryngologist and may represent a diagnostic challenge. The differential diagnosis of cystic anterior triangle neck lumps include thyroid cysts, thymic cysts, thyroglossal duct cysts, branchial cleft cysts, bronchogenic cysts, and lymphangiomas. Parathyroid cysts (PC) are a rare clinical entity in routine practice, representing 0.5–1% of parathyroid lesions and <1% of neck masses [1]. They were first described in 1880 by Sandstorm and can be divided into functioning and nonfunctioning depending on their ability to secrete parathyroid hormone (PTH) [2]. Physical examination is nonspecific and preoperative diagnosis is usually difficult.

We report a case of a nonfunctioning PC and present the clinicopathologic findings and diagnostic challenge, drawing attention to the importance of including it in the differential diagnosis of neck lumps.

## 2. Case Report

A 44-year-old lady presented to our otolaryngology department with a five-month history of an anterior neck swelling which fluctuated in size. She complained only of fatigue, with no symptoms of thyroid or parathyroid dysfunction or local compressive symptoms. The mass was approximately 5 × 5 cm and nontender with a soft, smooth consistency in the absence of palpable neck nodes. Her weight was stable and vital signs were within normal limits. Thyroid function tests including triiodothyronine, thyroxine, and thyroid stimulating hormones were within the reference ranges and the serum adjusted calcium was 2.37 mmol/L.

**Figure 1:**
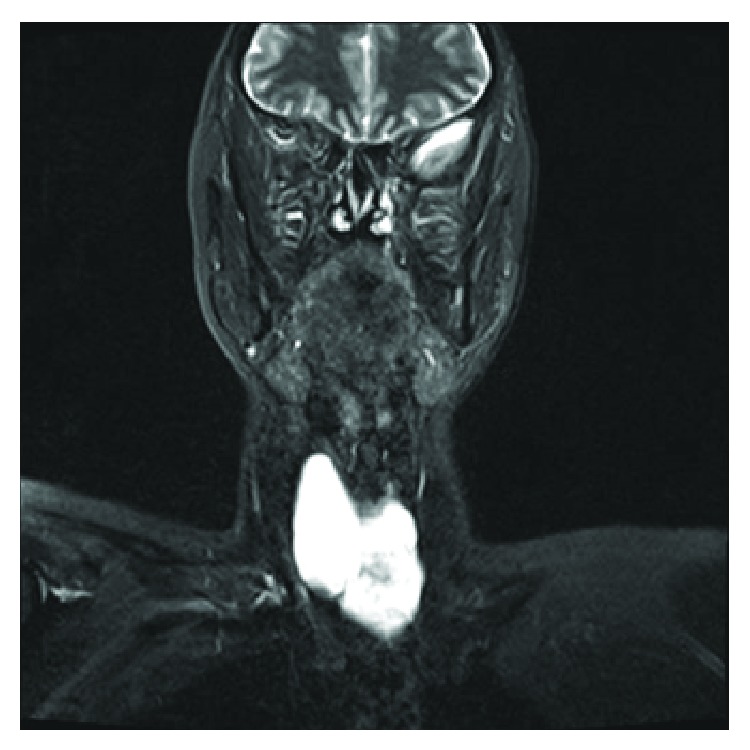
T2-weighted coronal MRI demonstrating a pyramidal shaped cystic lesion in continuity with the lower portions of both thyroid lobes.

**Figure 2:**
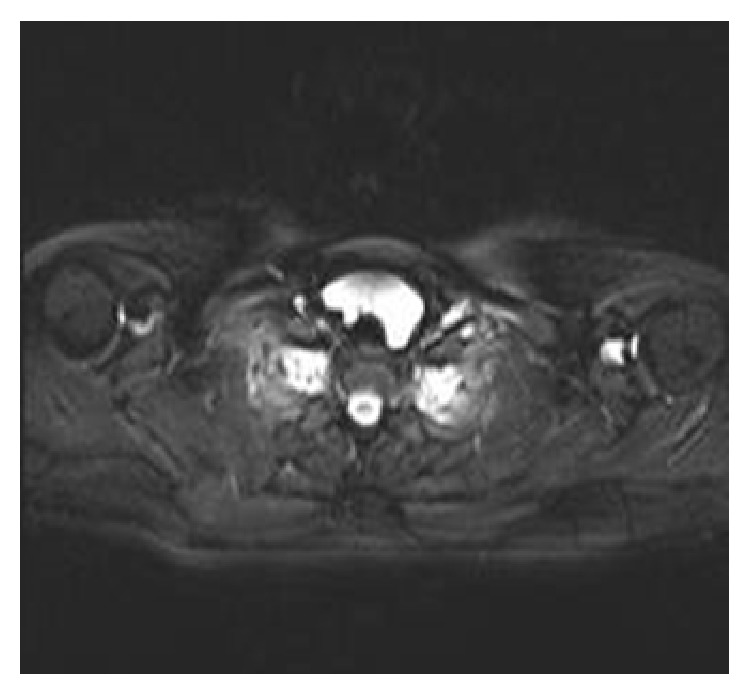
T2-weighted axial MRI showing the lesion's pretracheal position.

Fine-needle aspiration (FNA) of the swelling revealed clear fluid with cytology demonstrating “scanty foamy histiocytes.” Fluid PTH level was not measured. Ultrasound scan (USS) reported a 7.5 cm cystic lesion inferior to the thyroid with no internal vascularity. Appearances were not consistent with a thyroid cyst and ultrasonography failed to determine the origin of the cystic swelling. Magnetic resonance imaging (MRI) was employed in an attempt to further delineate the lesion. This revealed a pretracheal 5.3 cm × 2.9 cm (transverse × anteroposterior) × 5.8 cm (craniocaudal) pyramidal-shaped, bilobed cystic lesion in continuity with the lower portions of both thyroid lobes (Figures 1 and 2). Differential diagnosis at this stage included a thymic cyst extending into the neck.

Despite the above investigations, the diagnosis remained unclear. With the risk of the swelling increasing in size combined with the small potential risk of malignancy, it was felt that surgical exploration was warranted.

**Figure 3:**
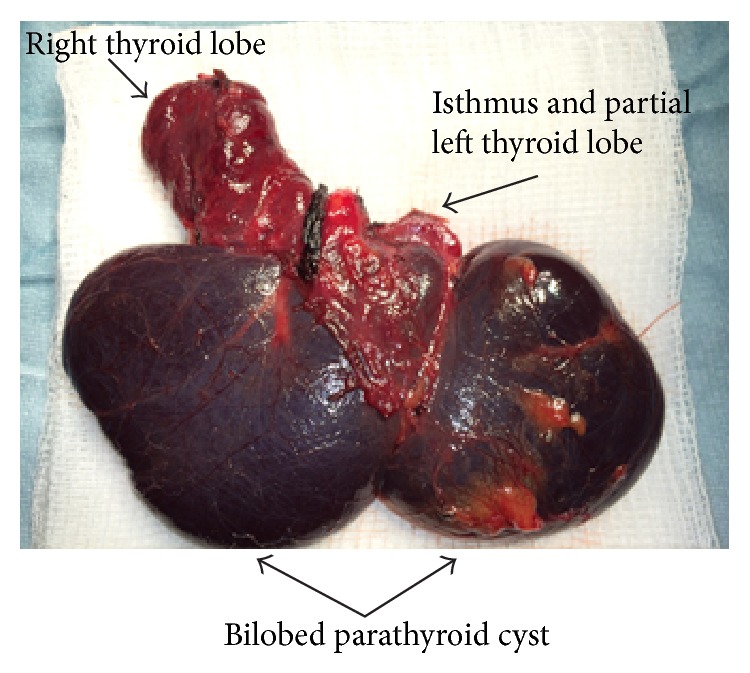
Parathyroid cyst intimately related to the inferior aspect of the thyroid gland, arising from the right inferior parathyroid gland.

Intraoperatively a thin walled bilobed cyst arising from the inferior thyroid, based predominantly on the right side, was visualised and an extended right hemithyroidectomy was performed (right lobectomy, isthmusectomy, and partial left lobectomy). A significant portion of the left thyroid lobe was retained to preserve thyroid function. Simple cyst excision was not deemed to be appropriate as the diagnosis was unclear at the time of surgical exploration and the lesion was intimately related to both thyroid lobes. Histopathological sections demonstrated a 7 cm cystic abnormality with a fibrofatty wall lined by a low columnar epithelium arising from the right inferior parathyroid gland. Parathyroid tissue with no cellular atypia was identified focally within the cyst wall. The thyroid gland was unremarkable.  Figure 3 demonstrates the operative specimen.

The patient made an uneventful recovery and was discharged on postoperative day one. Serum thyroid function tests, parathyroid hormone, and calcium remained within the reference ranges in the immediate postoperative period. The patient remains asymptomatic with no recurrence or serum biochemical abnormalities at 6-month follow-up.

## 3. Discussion

Eighty percent of PC are orthotopic, solitary, and nonfunctioning, presenting as asymptomatic neck swellings anterior to the inferior pole of the thyroid gland [3]. Embryologically, the superior parathyroid glands are closely related to the thyroid gland whereas the inferior parathyroid glands are intimately related to the thymus. This becomes clinically important because PC may be ectopic and occur anywhere in the neck, mimicking a thyroid or mediastinal lesion [3]. Indeed, the clinical presentation of PC frequently resembles thyroid nodules leading the clinician to mistakenly believe that thyroid disease is involved.

Nonfunctioning cysts are more common in women (1 : 2.5 male-to-female ratio), usually arising in the fourth or fifth decade, with 70% originating from the inferior parathyroid glands [4]. However, the 10% of PC that are associated with hypercalcaemia (functioning cysts) demonstrate a male preponderance and invariably contain elevated C-terminal PTH (C-PTH) levels [5]. The symptoms of nonfunctioning cysts are limited to those caused by compression of neighbouring structures whereas the problems associated with functioning cysts are related to excessive secretion of PTH and subsequent hyperparathyroidism and hypercalcaemia [3, 6].

The pathogenesis is unclear, but several theories have been proposed. The cysts may result from a vestigial remnant of the third or fourth brachial cleft or from the persistence of Kürsteiner canals [7]. Other theories propose that PC develop from the infarction and degeneration of a parathyroid adenoma or from the coalescence of multiple microcysts in normal parathyroid tissue [3]. Microcysts are a common finding in the ageing parathyroid glands and autopsy material reveals that up to 50% of asymptomatic glands contain microcysts [6]. Conversely, macrocysts are uncommon and always warrant clinical investigation.

Assessment of PC includes a thorough head and neck examination followed by FNA and USS. Fine-needle aspiration often reveals clear, colourless cystic fluid and a preoperative diagnosis of PC can be confirmed by demonstrating raised C-PTH levels, irrespective of whether the cyst is functioning or nonfunctioning [8, 9]. It must be kept in mind that levels of C-PTH do not correlate with the functional activity of the lesion. However, C-PTH testing of FNA fluid represents a valuable diagnostic tool and should be performed in all cystic neck swellings, especially when the diagnosis is in doubt. It is recommended that C-PTH is measured from cystic fluid rather than intact PTH because nonfunctioning parathyroid cysts can produce large quantities of PTH which is rapidly degraded into the biologically inactive C-terminal peptide [8]. Therefore, parathyroid cysts may have a relatively low intact PTH level, underestimating their ability to produce PTH and resulting in a false negative diagnosis [8]. Conversely, FNA of thyroid cysts classically reveals a cloudy, gelatinous, or blood-stained aspirate negative for C-PTH [10]. Despite this, without aspirate biochemical analysis, diagnosis often remains in doubt as the presence of turbid or coloured fluid does not exclude the diagnosis of PC as fluid from a functioning PC may occasionally be yellow or brown due to haemosiderin in an infarcted parathyroid adenoma [3].

Although imaging is a useful part of the preoperative assessment of such lesions, there are no specific radiological methods for differentiating parathyroid cysts from other cystic neck lesions. Ultrasonography may reveal a nonspecific cystic structure. Ujiki et al. showed that PC were seldom correctly diagnosed by USS suggesting that this modality cannot differentiate parathyroids from other cystic neck lesions [11]. A major limitation of their study was that USS was only performed in 50% of cases, so selection bias may have been introduced which could account for the low sensitivity reported. Current techniques for head and neck imaging such as computerised tomography (CT) and MRI may demonstrate the cystic component of these lesions and help to visualise the relationship with adjacent tissues. These imaging modalities are particularly useful in the presence of substernal extension or compressive symptoms. They may also be employed when a diagnosis cannot be reached using FNA and USS, if malignancy is suspected, or for preoperative planning. Radioiodine thyroid scanning has been utilised and may suggest a nonfunctioning cold nodule. A study by Hughes et al. showed that T1-201-Tc-99m pertechnetate subtraction scintigraphy, CT, and USS were unable to distinguish parathyroid cysts from thyroid nodules and failed to demonstrate the exact origin of cystic neck masses [12].

Despite the ability to detect PC preoperatively, many parathyroid cysts are only diagnosed postoperatively. For those identified preoperatively, treatment can be controversial. Although rare, two cases of parathyroid cyst carcinoma have been reported in the literature [13, 14]. Both of these cases have arisen in functioning cysts, with no report of carcinoma in nonfunctioning cysts. Therefore, the primary management of functioning cysts (or nonfunctioning cysts with compressive symptoms) is surgical excision to confirm diagnosis, exclude malignancy, and remove the swelling, according to well-established guidelines for patients with hyperparathyroidism [15, 16].

In contrast, therapeutic ultrasound-guided FNA is favoured by some authors as the primary management of nonfunctioning PC [17]. This safe and easy technique allows a certain diagnosis in most cases, as well as being therapeutic. Clark was the first to describe aspiration alone for the treatment of PC [18]. He reported that these cysts are usually benign and hence may be treated nonoperatively by aspiration alone; however cyst recurrence is problematic. Multiple studies have evaluated the success rate of simple aspiration of PC alone, with therapeutic success rates between 33 and 92% [5, 19–22]. In a case series by Ujiki et al. 75% of patients required multiple aspirations for recurrence; the first repeat aspiration is always within one year [11]. Sung et al. treated 12 patients with nonfunctioning PC by simple aspiration; 4 were successful, while 8 patients had a recurrence [17]. In an attempt to avoid surgical management, the authors treated the recurrent cysts by injection of an ethanol volume that was <50% of the aspirate volume. In this series, there were no significant complications reported in any of the 12 cases described although 2 patients did require a second alcohol ablation because of insufficient volume reduction. This represents a significant primary failure rate (17%). Furthermore, extracystic fibrosis and recurrent laryngeal nerve palsy with subsequent vocal cord palsy following the use of sclerosing agents have been reported and the long-term efficacy and success of this treatment remain unclear [23, 24]. Therefore, sclerotherapy is not uniformly accepted at present.

Prinz et al. propose that the initial treatment of nonfunctioning PC should be needle aspiration, followed by tetracycline sclerosis after a second recurrence [19]. If cyst aspiration or sclerosis fails, or in the presence of compressive symptoms, surgical management should be considered. However, McCoy et al. argue that first-line management of all PC, irrespective of their functional status, is surgical excision as the morbidity of surgery in specialist centres is low [15].

Microscopically, nonfunctioning PC consist of a smooth internal layer with a thin membranous layer. The cyst wall is formed by a single layer of cuboidal or columnar epithelium that stains positive for glycogen [25]. If the cyst is discovered at the time of surgery, several macroscopic features may help to distinguish it; a smooth, shiny, semitransparent thin cyst wall that is usually loosely attached to the thyroid is easily dissected free from the thyroid and surrounding tissue. Intraoperative cyst rupture should be avoided to prevent parathyromatosis [15]. Serum calcium levels should be monitored postoperatively as hypocalcaemia may occur following parathyroid cyst excision. Patients with large cysts (≥4 g) are at higher risk of symptomatic hypocalcaemia and should be managed expectantly [15].

## 4. Conclusion

Parathyroid cysts are uncommon benign neck lesions. They may be functioning or nonfunctioning and lack characteristic clinical and radiological features, often misleading the diagnosis. Fine-needle aspiration and cystic fluid C-PTH levels represent a valuable diagnostic tool and should be performed in all cystic neck swellings, especially when the diagnosis is in doubt. Management of nonfunctioning cysts remains controversial, and the effectiveness of relatively recently described techniques of needle aspiration or sclerosant injection as an alternative to definitive surgical excision is variable. Parathyroid cysts must be included within the differential diagnosis of cystic neck swellings or in patients with hyperparathyroidism or hypercalcaemia.
